# Prediction of post-PCI angina risk using machine learning-based magnetocardiography model

**DOI:** 10.3389/fcvm.2026.1750482

**Published:** 2026-04-20

**Authors:** WenLong Wang, LiNa Wang, FaMing Ding, ZhiXin Wang, ShiLong Cao, QingMin Ji, MengFan Hu, JianGuo Cui, Dong Wang

**Affiliations:** Department of Cardiology, Binzhou Medical University Hospital, Binzhou, Shandong, China

**Keywords:** cTnI, LDL, machine learning, magnetocardiogram, NT-ProBNP, percutaneous coronary intervention, Seattle Angina Questionnaire

## Abstract

**Background:**

Recurrent angina after percutaneous coronary intervention (PCI) impairs quality of life and poses a clinical challenge. Magnetocardiography (MCG), as a noninvasive tool, its role in predicting symptomatic outcomes post-PCI remains undefined.

**Objective:**

This study sought to develop and validate a machine learning-based MCG model, both alone and combined with clinical biomarkers, to predict angina after PCI.

**Methods:**

110 patients with coronary artery disease undergoing successful PCI were included. MCG was performed pre-PCI and within 72 h post-PCI. MCG was performed to derive quantitative scores for all patients both prior to and following percutaneous coronary intervention (PCI). Angina status was assessed within 3 months using the Seattle Angina Questionnaire-Angina Stability (SAQ-AS) and -Angina Frequency (SAQ-AF) domains. Based on SAQ-AS scores, patients were stratified into an AS-negative group (*n* = 105, mean age 62 ± 8 years, 59% male) and an AS-positive group (*n* = 5, mean age 65 ± 15 years, 40% male). Similarly, SAQ-AF scores categorized patients into an AF-negative group (*n* = 101, mean age 63 ± 8 years, 60.4% male) and an AF-positive group (*n* = 9, mean age 60 ± 12 years, 33.3% male). Serum biomarkers, including low-density lipoprotein cholesterol (LDL-C), cardiac troponin I (cTnI), and N-terminal pro-B-type natriuretic peptide (NT-proBNP), were measured. An MCG score was measured for each patient both pre- and post-PCI. An MCG model and a combined model integrating MCG with biomarkers were developed using multivariable logistic regression. Performance was evaluated by the area under the receiver operating characteristic curve (AUC), sensitivity, and F1-score. Prediction models were visualized using nomograms.

**Results:**

MCG score decreased from 0.783 pre-PCI to 0.616 post-PCI. The combined model demonstrated superior discriminatory ability for SAQ-AS. For SAQ-AF, the combined model achieved higher sensitivity (0.663 vs. 0.605), F1-score (0.797 vs. 0.754), and AUC (0.813 vs. 0.801) compared to MCG alone. The nomogram provided broader risk stratification (SAQ-AS: 0.7–0.9; SAQ-AF: 0.5–0.9). Calibration was satisfactory (Hosmer–Lemeshow *p* > 0.05).

**Conclusion:**

A machine learning-based MCG model effectively predicts post-PCI angina risk. Integrating MCG with clinical biomarkers enhances risk stratification, offering a noninvasive strategy for identifying high-risk patients.

## 1 Introduction

Percutaneous coronary intervention (PCI) serving as a cornerstone in the management of coronary artery disease (CAD) to alleviate symptoms of myocardial ischemia and improve quality of life (1–3). Despite successful anatomical revascularization, a significant number of patients experience persistent or recurrent angina, a condition associated with impaired functional status, increased healthcare utilization, and worse long-term outcomes (4–6).

Therefore, accurate and timely assessment of post-PCI efficacy is essential for identifying patients with residual ischemia. However, current modalities for post-procedural evaluation each present significant limitation. While coronary angiography (CAG) remains the anatomical gold standard, it is restricted to visualizing epicardial stenosis and provides no functional assessment of microvascular dysfunction (5, 7). Its invasive nature also precludes serial evaluation due to associated vascular risks (8, 9).

Cardiac magnetic resonance (CMR), though capable of evaluating microvascular function and viability, requires gadolinium-based contrast agents—problematic in renal impairment and raising concerns about long-term intracranial deposition (10, 11). Additionally, extended scan times (45–60 min) challenge its utility in heart failure patients. Myocardial perfusion imaging with SPECT entails substantial radiation exposure (10–15 mSv per session), limiting repeated use (12), while echocardiography remains constrained by acoustic windows and operator dependency, often yielding suboptimal images in obese patients (13).

The Seattle Angina Questionnaire (SAQ), a validated patient-reported outcome measure, has become a critical tool for quantifying this symptomatic burden, with its Angina Stability (SAQ-AS) and Angina Frequency (SAQ-AF) domains offering robust metrics for clinical evaluation (14, 15).

Magnetocardiography (MCG) represents a breakthrough technology with distinct advantages for cardiac assessment, including high sensitivity, rapid acquisition, operational simplicity, and absence of radiation exposure (16, 17). MCG can detect weak magnetic generated by cardiac electrical activity. Due to its unique sensitivity to alterations in intracellular ionic currents—particularly those disturbed by ischemia—MCG can identify functional abnormalities that are often undetectable by conventional electrocardiography (6).The unique physical principles of magnetocardiography confer notable advantages. It is free from signal attenuation, enables non-contact detection, and allows magnetic fields to penetrate human tissues without distortion, thereby facilitating high-fidelity recording of electrical activity. With no associated radiation exposure, MCG is particularly well-suited for repeated follow-up assessments (each requiring ≤10 min). Furthermore, its independence from contrast agents eliminates any contraindications for use in patients with impaired renal function (18, 19).

Previous studies have established the diagnostic accuracy of MCG in CAD (20). However, the existing clinical evidence regarding the use of MCG to assess post-PCI angina remains limited (19). Consequently, we conducted this prospective study to validate whether a machine learning model based on MCG could effectively predict post-PCI angina, as defined by the Angina Stability (AS) and Angina Frequency (AF) scores of the SAQ.

## 2 Methods

### 2.1 Study design and population

This was a prospective, single-center, cross-sectional study that obtained approval from the Binzhou Medical University Hospital Ethics Committee (Approval No.: KYLL-120). All participants were fully informed of the study objectives and provided written informed consent prior to enrollment. The study consecutively enrolled hospitalized patients from August 2024 to July 2025. A total of 587 patients presenting with chest pain and scheduled for CAG and PCI were recruited. MCG data were collected pre-procedure and within 72 h post-procedure. Follow-up assessments were conducted using the SAQ-AS and SAQ-AF within 3 months after PCI.

Four hundred and seventy-seven patients were excluded based on the following criteria: (1) age less than 18 years; (2) New York Heart Association (NYHA) functional class IV; (3) comorbid conditions including bundle branch blocks, premature ventricular contractions, atrial fibrillation, other arrhythmias, or other systemic diseases; (4) did not undergo PCI or MCG examination; (5) poor-quality MCG images deemed unsuitable for analysis; and (6) failure to complete the Seattle Angina Questionnaire.

**Figure 1:**
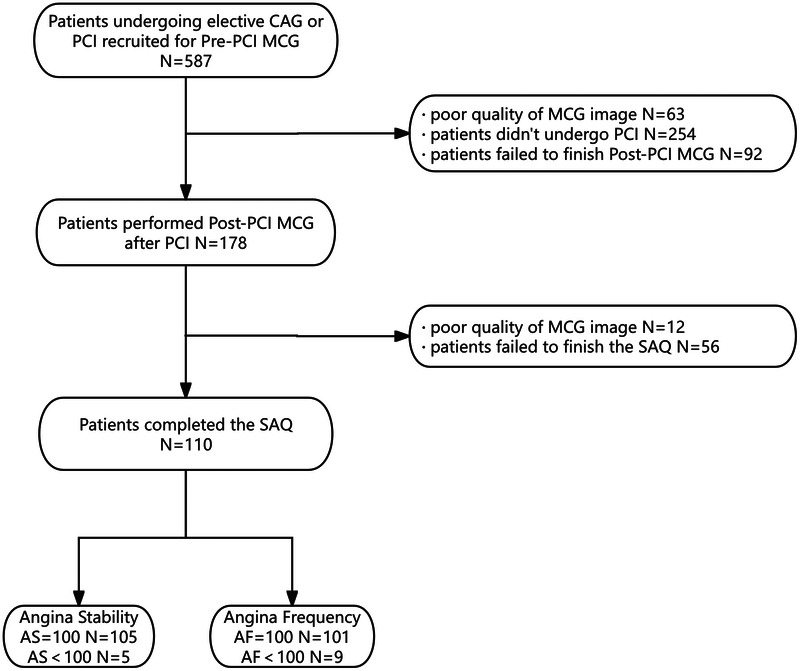
Flowchart of patients enrolled. CAG, coronary angiography; PCI, percutaneous coronary intervention; MCG, magnetocardiography; SAQ, Seattle Angina Questionnaire.

Consequently, 110 patients constituted the final study cohort. Based on SAQ-AS scores, patients were categorized into an AS-negative group (*n* = 105, mean age 62 ± 8 years; 59% male) and an AS-positive group (*n* = 5, mean age 65 ± 15 years; 40% male). Similarly, according to SAQ-AF scores, patients were divided into an AF-negative group (*n* = 101, mean age 63 ± 8 years; 60.4% male) and an AF-positive group (*n* = 9, mean age 60 ± 12 years; 33.3% male). The study flow chart is presented in Figure 1.

Upon enrollment, all patients underwent MCG examination within 48 h prior to CAG. For patients who subsequently underwent PCI, a second MCG assessment was performed within 72 h after the procedure. The MCG data were collected and processed by a professionally trained technician who was blinded to the study endpoint outcomes.

Detailed clinical information was retrospectively obtained from electronic medical records at admission and discharge. Additional laboratory biochemical parameters and echocardiographic data, acquired using standardized methodologies, were systematically collected for all included patients.

### 2.2 MCG acquisition and analysis

Cardiac magnetic fields were acquired using a magnetocardiography system (Distin OP 300, Shanghai MEDI Instruments Ltd., Shanghai, China). This system utilizes highly sensitive SERF atomic magnetometers as magnetic sensors, comprising 14 detection channels. It achieves a magnetic field measurement sensitivity exceeding 40 fT/√Hz with a measurement bandwidth of 0.05 Hz to 100 Hz.

The processed magnetocardiographic signals, after noise suppression and multi-cycle averaging, were used to generate spatial waveform maps, isofield maps, and pseudo-current density maps for the analysis of the cardiac magnetic field and assessment of patient health status.

**Figure 2:**
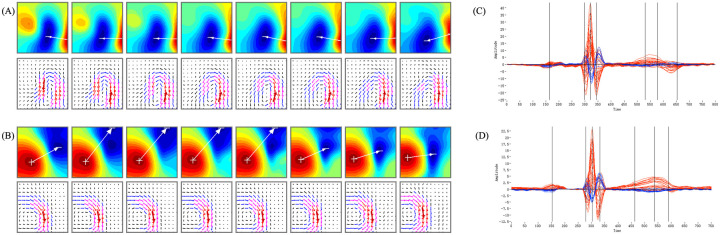
MCG maps of a patient pre- and post-PCI. **(A)**: MFM and PCDM of a patient pre-PCI; **(B)**: MFM and PCDM in the same patient post-PCI; **(C)**: Butterfly plots recorded simultaneously with **(A)**; **(D)**: Butterfly diagram recorded simultaneously with **(B)**.

Prior to MCG imaging, patients were required to remove all ferromagnetic metallic objects (e.g., jewelry, eyeglasses, dentures) from their body. Patients were positioned supine on the examination table and instructed to maintain quiet breathing. The sensor array was precisely positioned by the operator directly above the anterior chest region. Using a non-contact scanning technique at a sampling frequency of 1,000 Hz, data were sequentially captured from three scanning points, acquiring cardiac magnetic signal data from 36 detection points covering a 250 mm × 250 mm area over the chest surface. Figure 2 presents the magnetocardiographic (MCG) maps of a patient before and after percutaneous coronary intervention (PCI). Panels A and B show the T-wave isomagnetic field maps (MFM) (on the top row) and pseudo-current density maps (PCDM)(at the bottom) pre- and post-PCI, respectively; Panels C and D display the corresponding butterfly plots. The MFM and PCDM maps revealed an abnormal T-wave angle pre-PCI, which normalized after the intervention. Consistently, the butterfly diagram revealed a biphasic T-wave morphology prior to PCI, which reverted to a monophasic pattern after revascularization.

### 2.3 Myocardial ischemia assessment

In this study, an AI model was employed to evaluate myocardial ischemia in enrolled patients with coronary artery disease. The model was trained and validated using data from 2,426 patients with coronary artery disease collected from multiple healthcare institutions, with diagnosis of ischemic heart disease confirmed by coronary angiography showing stenosis exceeding 70% in at least one major vessel. For clinical application, the model outputs were presented as myocardial ischemia probability scores, with an optimal diagnostic threshold (21).

All MCG-recorded ischemia scores were analyzed, comparing differences between pre- and post-PCI measurements. Receiver Operating Characteristic (ROC) curves were generated using post-procedural MCG data alone and in combination with clinical information, with the SAQ-AS and SAQ-AF dimensions serving as reference standards.

### 2.4 Assessment of coronary artery stenosis and percutaneous coronary intervention

All patients underwent CAG in the catheterization laboratory. Intracoronary nitroglycerin was administered prior to the procedure to prevent vascular spasm. Diagnostic CAG was performed using standard techniques, and digital images from multiple angiographic projections were recorded.

The degree of vascular stenosis was assessed by visual estimation from two experienced cardiologists. The operators also determined whether revascularization was clinically indicated for each patient. The final label for stenosis severity was determined by averaging the assessment results from the two physicians. The evaluation included both the quantitative stenosis severity and the clinical need for further intervention.

PCI procedures were performed in accordance with the guidelines established by the American Heart Association and the Chinese Society of Cardiology (22).

### 2.5 Data analysis

Data analysis was performed using SPSS 27.0 and R statistical software. Measurement data are presented as mean and standard deviation. Normally distributed variables are expressed as mean ± standard deviation (x¯ ± s) and compared using independent samples t-test. Non-normally distributed variables are expressed as median and interquartile range (IQR) and compared using the Mann–Whitney U non-parametric test. Categorical data are presented as frequencies and percentages, with intergroup comparisons conducted using the chi-square (*χ*^2^) test. Receiver operating characteristic (ROC) curves were plotted to calculate the model's area under the curve (AUC), along with its sensitivity, specificity, accuracy, and F1 score. A *P*-value < 0.05 was considered statistically significant.

## 3 Results

### 3.1 Participant baseline characteristics

Baseline characteristics of the patients are summarized in Table 1. No significant differences were observed in demographic or clinical characteristics between groups when stratified by SAQ-AS or SAQ-AF criteria. Notably, levels of NT-proBNP and cTnI were lower in the AS-negative and AF-negative groups compared to the AS-positive and AF-positive groups, consistent with the clinical manifestations of myocardial ischemia. Furthermore, the left anterior descending (LAD) artery was the most frequently involved vessel in both the AS and AF scoring systems.

**Table 1:** Demographic characteristics and clinical indicators of the study patients (*n* = 110).

Parameters	Angina stability			Angina frequency		
	AS < 100 (*n* = 5)	AS = 100 (*n* = 105)	*P* value	AF < 100 (*n* = 9)	AF = 100 (*n* = 101)	*P* value
Age, yrs	65 ± 15	62 ± 8	0.551	60 ± 12	63 ± 8	0.989
Male	2 (40%)	62 (59%)	0.475	3 (33.3%)	61 (60.4%)	0.99
Height, cm	160 ± 8	165 ± 7	0.941	159 ± 6	165 ± 7	0.997
Weight, kg	65.8 ± 8.5	71.1 ± 10.3	0.941	64.6 ± 9.7	71.4 ± 10.2	0.998
BMI	24.75 ± 1.46	25.96 ± 2.84	0.981	24.88 ± 3.42	26.00 ± 2.75	0.997
History of diabetes	0 (0%)	27 (25.7%)	0.998	0 (0%)	27 (26.7%)	0.985
History of hypertension	4 (80%)	74 (70.5%)	0.565	8 (88.9%)	70 (69.3%)	0.996
History of PCI	0 (0%)	27 (25.7%)	0.998	1 (11.1%)	26 (25.7%)	0.994
Prior complete revascularization	4 (80%)	75 (71.4%)	0.691	8 (88.9%)	71 (70.3%)	0.993
LVEF, %	61.25 ± 0.96	59.04 ± 5.48	0.254	60.75 ± 1.83	58.97 ± 5.59	0.995
TC, mmol/L	3.21 ± 0.92	4.78 ± 1.56	0.966	4.24 ± 1.47	4.77 ± 1.57	0.995
LDL, mmol/L	2.06 ± 0.78	2.88 ± 1.07	0.756	2.73 ± 1.14	2.86 ± 1.08	0.994
HDL, mmol/L	0.935 ± 0.08	1.17 ± 0.29	0.224	1.14 ± 0.24	1.16 ± 0.30	0.995
NT-proBNP, pg/mL	120.00 (60.63–207.50)	73.48 (52.88–343.00)	0.536	122.30 (63.11–207.55)	66.42 (31.52–178.00)	0.993
cTnI, ng/mL	0.10 ± 0.15	0.71 ± 3.62	0.91	0.06 ± 0.11	0.75 ± 3.72	0.999
Target Vessel						
RCA	0 (0%)	32 (30.5%)	0.999	1 (11.1%)	31 (30.7%)	0.988
LAD	4 (80%)	59 (56.2%)	1	7 (77.8%)	56 (55.4%)	0.996
LCX	1 (20%)	30 (28.6%)	0.998	2 (22.2%)	29 (28.7%)	1
Diseased vessel						
RCA	0 (0%)	45 (42.9%)	0.999	1 (11.1%)	44 (43.6%)	0.988
LAD	4 (80%)	77 (73.3%)	0.998	7 (77.8%)	74 (73.3%)	0.997
LCX	2 (40%)	49 (46.7%)	0.471	3 (33.3%)	48 (47.5%)	0.998
Number of diseased vessels			0.215			0.109
Single-vessel disease	4 (80%)	57 (54.3%)		7 (77.8%)	54 (53.5%)	
Double-vessel disease	1 (20%)	30 (28.6%)		2 (22.2%)	29 (28.7%)	
Triple-vessel disease	0 (0%)	18 (17.1%)		0 (0%)	18 (17.8%)	

All values presented as *n* (%) or mean ± SD. AS, Angina stability; AF, Angina frequency; BMI, body mass index; LVEF, left ventricular ejection fraction; TC, total cholesterol; LDL, low-density lipoprotein; HDL, high-density lipoprotein; NT-proBNP, N-terminal pro-B-type natriuretic peptide; cTnI, cardiac troponin I; RCA, right coronary artery; LAD, left anterior descending artery; LCX, left circumflex artery.

### 3.2 Changes in MCG-derived myocardial ischemia scores following revascularization

**Figure 3:**
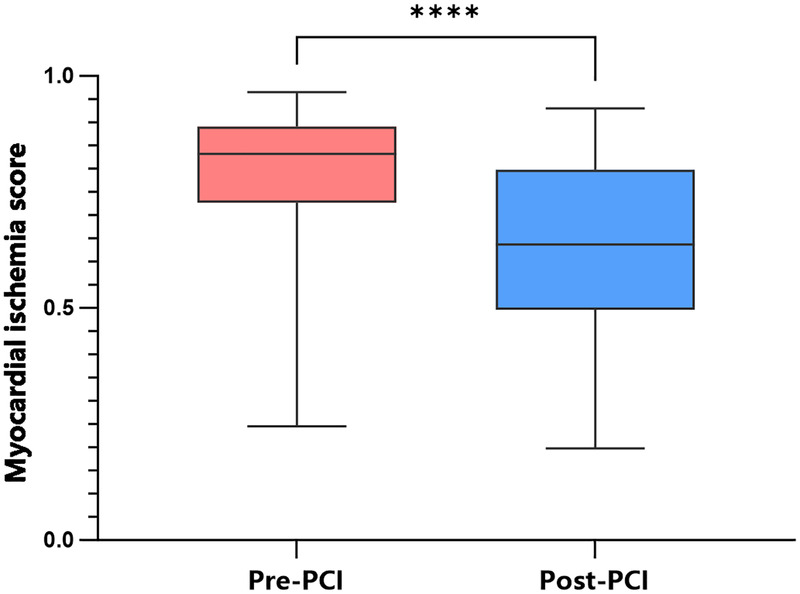
Box plot of myocardial ischemia scores. The box plot demonstrates a significant decrease in the median myocardial ischemia score after PCI compared to the pre-operative level (*P* < 0.05), indicating that PCI treatment leads to a highly significant improvement in myocardial ischemia.

The mean MCG-derived score decreased from 0.783 before PCI to 0.616 after PCI. The pre-procedural group (red) exhibited a higher median score, indicating a state of relatively severe myocardial ischemia at baseline. In contrast, the post-procedural group (blue) shows a clear downward shift, with its median significantly lower than the pre-procedural group. This visually reflects a general improvement in myocardial ischemia following revascularization (Figure 3).

### 3.3 The diagnosis performance of MCG model and combined model with clinical parameters

Multivariable analysis of the subjects’ biochemical indicators identified that LDL, cTnI, and NT-proBNP were independent predictors of post-PCI AS positivity (ORs of 2.924, 1.088, and 1.001, respectively; Table 2).

**Table 2:** Multivariate logistic regression of predictors for AS.

Term	B	SE	Wald *χ*²	P	OR	95% CI
(Intercept)	4.587	2.213	4.298	0.038	98.219	
LDL	1.073	3.447	0.097	0.756	2.924	0.003–2,510.489
cTnI	0.085	0.752	0.013	0.91	1.088	0.249–4.748
NT-proBNP	0.001	0.001	0.384	0.536	1.001	0.999–1.002
TC	−0.114	2.642	0.002	0.966	0.893	0.005–158.408
HDL	−3.416	2.807	1.481	0.224	0.033	0–8.049

AS, Angina stability; LDL, low-density lipoprotein; cTnI, cardiac troponin I; NT-proBNP, N-terminal pro-B-type natriuretic peptide; TC, total cholesterol; HDL, high-density lipoprotein.

**Figure 4:**
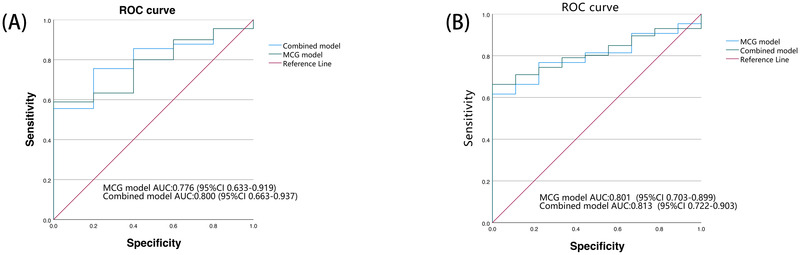
Performance of predictive value of diagnosis models. **(A)**: Receiver operating characteristic (ROC) curves of the MCG model and the combined model for predicting postoperative AS episodes. The areas under the curve (AUC) were 0.776 (95% CI: 0.633-0.919) for the MCG model and 0.800 (95% CI: 0.663-0.937) for the combined model. AUC: area under the curve; MCG: magnetocardiography. **(B)**: Receiver operating characteristic (ROC) curves of the MCG model and the combined model for predicting postoperative AF episodes. The areas under the curve (AUC) were 0.801 (95% CI: 0.703-0.899) for the MCG model and 0.813 (95% CI: 0.722-0.903) for the combined model.

These three clinical parameters were integrated with the MCG model to construct a combined Model. The results, as shown in Figure 4A, Table 3, For predicting AS, the MCG model achieved marginally higher Sensitivity (0.589 vs.0.556) and F1 scores (0.741 vs.0.715) compared to the Combined Model. However, the Combined Model demonstrated a higher AUC than the MCG model, indicating improved the model's overall discriminative ability.

**Table 3:** MCG model and combined model performance estimation for AS.

Indicator	MCG model	Combined model
Cutoff	0.580	0.970
F1 Scores	0.741	0.715
Sensitivity	0.589	0.556
Specificity	1.000	1.000
PPV	1.000	1.000
NPV	0.981	0.979
ACC	0.981	0.980

MCG, magnetocardiography; AS, Angina stability; PPV, positive predictive value; NPV, negative predictive value; ACC, accuracy.

Nomograms were plotted to visualize the two logistic regression models for predicting postoperative AS (Supplementary Figure S1). A higher total point score, calculated as the sum of points assigned to each predictor, indicates a greater risk of postoperative AS. In Supplementary Figure S1A, the MCG model predicts post-procedural AS risk with probabilities concentrated in a narrow, high-risk range (0.8–0.9), making it difficult to perform finer sub-stratification within this high-risk population. Supplementary Figure S1C shows the Combined model, which incorporates LDL, cTnI, and NT-proBNP into the MCG model. The inclusion of these three key clinical biomarkers provides complementary information from three distinct yet critical pathophysiological perspectives: lipid metabolism, myocardial injury, and cardiac wall stress. This Combined model predicts post-procedural AS risk over a wider probability range (0.7–0.9), offering better discriminative ability and supporting more precise clinical decision-making.

Both models exhibit excellent calibration, and their predicted risks are highly reliable. The Hosmer-Lemeshow test yielded *P*-values of 0.760 for the MCG model and 0.654 for the Combined model, both well above the significance level of 0.05, demonstrating that the nomograms have good predictive accuracy (Supplementary Figures S1B, D).

For predicting AF, LDL, cTnI, and NT-proBNP were also independent predictors (ORs: 1.627, 1.523, and 1.001, respectively; Table 4). The combined model outperformed the MCG model, with higher sensitivity (0.663 vs. 0.605), F1-score (0.797 vs. 0.754), accuracy (0.972 vs. 0.968), and AUC (0.813 vs. 0.801; Figure 4B, Table 5).

**Table 4:** Multivariate logistic regression of predictors for AF.

Term	B	SE	Wald χ²	P	OR	95% CI
(Intercept)	3.991	1.748	5.21	0.022	54.088	
LDL	0.486	2.359	0.043	0.837	1.627	0.016–165.816
cTnI	0.421	1.714	0.06	0.806	1.523	0.053–43.861
NT-proBNP	0.001	0.002	0.211	0.646	1.001	0.997–1.004
TC	−0.098	1.799	0.003	0.956	0.906	0.027–30.835
HDL	−2.367	1.99	1.415	0.234	0.094	0.002–4.633

AF, Angina frequency; LDL, low-density lipoprotein; cTnI, cardiac troponin I; NT-proBNP, N-terminal pro-B-type natriuretic peptide; TC, total cholesterol; HDL, high-density lipoprotein.

**Table 5:** MCG model and combined model performance estimation for AF.

Indicator	MCG Model	Combined Model
Cutoff	0.585	0.922
F1	0.754	0.797
Sensitivity	0.605	0.663
Specificity	1.000	1.000
PPV	1.000	1.000
NPV	0.966	0.971
ACC	0.968	0.972

MCG, magnetocardiography; AF, Angina frequency; PPV, positive predictive value; NPV, negative predictive value; ACC, accuracy.

The corresponding nomograms are presented in Supplementary Figure S2. The MCG model (Supplementary Figure S2A) concentrated predictions in a high-risk zone (>0.7), whereas the combined model (Supplementary Figure S2C) provided discrimination across a wider spectrum (0.5–0.9), effectively stratifying patients into moderate-, high-, and very-high-risk categories. Both models were well-calibrated (Hosmer-Lemeshow *P* = 0.873 and 0.221, respectively; Supplementary Figures S2B, D).

## 4 Discussion

In this prospective, single-center study, we demonstrated that machine learning-based magnetocardiography analysis effectively tracks functional improvement after PCI and provides reliable prediction of angina-related outcomes. The principal findings can be summarized as follows: First, the significant reduction in MCG-derived ischemia scores post-revascularization confirms the functional efficacy of PCI. Second, the MCG model demonstrated excellent performance in predicting angina as defined by the Seattle Angina Questionnaire (SAQ). Third, integration of MCG with clinical biomarkers (LDL-C, cTnI, and NT-proBNP) enhanced risk stratification across a broader probability spectrum. Finally, the developed nomogram offers a practical point-of-care tool that translates complex model outputs into readily interpretable individual risk probabilities, facilitating shared decision-making and guiding post-discharge management strategies. Both models demonstrated excellent calibration, as confirmed by the Hosmer-Lemeshow test, further reinforcing their predictive reliability.

Despite achieving anatomical success through stent deployment, persistent ischemia affects approximately 10%–30% of patients after PCI, often attributable to incomplete stent expansion, microcirculatory impairment, or plaque disruption (23–25). This persistent ischemia not only leads to ongoing symptoms but also significantly increases the risk of major adverse cardiac events to eliminate uncertainty and strengthen clarity. Conventional imaging modalities, while valuable for anatomical assessment, remain limited for early detection of these underlying pathophysiological mechanisms (26–28). Leveraging non-invasive magnetic field mapping, the MCG model enables the detection of subtle electrophysiological changes that reflect resolution of ischemia, thereby providing a functional assessment of revascularization success that marks a pivotal advance beyond anatomical evaluation alone. The marked reduction in MCG ischemia scores post-PCI robustly validates this technology's capacity to capture the recovery of myocardial electrophysiological function following successful revascularization. This observation aligns with fundamental pathophysiological principles wherein the relief of coronary stenosis normalizes transmembrane ionic currents; alterations directly reflected in the cardiac magnetic field (29).

Multivariate analysis confirmed that LDL, cTnI, and NT-proBNP serve as independent predictors for both SAQ angina stability and angina frequency following PCI (30–32). These biomarkers provide crucial information across three complementary pathophysiological dimensions: dysregulated lipid metabolism (LDL), myocardial microinjury (cTnI), and ventricular wall stress (NT-proBNP), thereby establishing a biological foundation for constructing the integrated model. Consequently, our findings extend MCG's application from a purely diagnostic modality to a dynamic tool for monitoring therapeutic response.

The magnetocardiography model developed in this study exhibited robust standalone performance. Its integration with biomarkers reflecting distinct pathological pathways-atherogenic lipids (LDL-C), cardiomyocyte necrosis (cTnI), and ventricular dysfunction (NT-proBNP)-yielded a more comprehensive and powerful predictive tool. The combined model demonstrated superior area under the curve (AUC) values and enhanced discriminatory capacity across the risk spectrum, as vividly illustrated in the nomogram, highlighting the synergistic value of integrating functional electrophysiological data with circulating biomarkers reflective of disease activity and burden.

The probabilities predicted by the MCG model were concentrated within a constrained high-risk range (SAQ-AS: 0.8–0.9; SAQ-AF: >0.7), limiting its capacity for fine stratification among high-risk subpopulations. In contrast, the combined model generated a broader probability distribution (SAQ-AS: 0.7–0.9; SAQ-AF: 0.5–0.9), effectively differentiating between moderate-, high-, and very-high-risk patients, thereby providing a more precise foundation for individualized treatment decisions. This methodology enables refined patient stratification, potentially identifying individuals at highest risk for symptomatic recurrence who may derive benefit from intensified secondary prevention and closer clinical surveillance. Applying these findings clinically, we implemented a triage system wherein patients showing clinical improvement were assigned to a green group for routine outpatient follow-up; those without improvement were categorized into a yellow alert group for prioritized assessment of key clinical indicators; and patients exhibiting deterioration were allocated to a red alert group for immediate intensive inpatient management.

## 5 Study limitations

Several limitations warrant acknowledgement. First, the single-center design coupled with a substantial number of excluded cases resulted in a relatively small final cohort—particularly in the AS-positive and AF-positive groups—which may compromise the generalizability of our findings and increase the risk of overfitting. External validation in larger, multicenter prospective cohorts is imperative. Second, the advanced MCG system employed in this study is currently confined to specialized centers, which may restrict its widespread applicability in the near term. Third, although the machine learning model used was trained on multi-institutional datasets, its inherent biases could affect performance and necessitate independent validation. Fourth, the follow-up period was limited to one year; longer-term studies are required to evaluate the durability of MCG-based predictions and their association with hard clinical endpoints, such as myocardial infarction or mortality. Finally, the assessment of angina was based on the SAQ, which, though validated, remains subjective and potentially influenced by non-cardiac factors.

## 6 Conclusion

In conclusion, our study confirms that magnetocardiography (MCG), particularly when integrated with key clinical biomarkers, provides a novel, non-invasive, and functional means of predicting symptomatic status following percutaneous coronary intervention (PCI). The significant post-procedural improvement in MCG scores underscore its sensitivity to ischemic burden, while the comprehensive predictive model offers a superior framework for risk stratification. These findings pave the way for a more personalized management approach in patients with coronary artery disease (CAD) post-revascularization. Future research should focus on validating this multiparametric strategy in broader populations and exploring its potential to guide therapeutic decision-making, ultimately aiming to improve the quality of life for patients with coronary artery disease.

## Data Availability

The original contributions presented in the study are included in the article/Supplementary Material, further inquiries can be directed to the corresponding authors.

## Supplementary material

Supplementary Figure 1 Nomogram construction and calibration curve of the nomogram. (A): Risk of postoperative AS predicted by the MCG model; (C): risk of postoperative AS predicted by the combined model, which was constructed by adding three biochemical indicators (LDL, cTnI, and NT-proBNP) to the MCG model. (B), (D): The ideal line represents perfect calibration. The apparent line indicates the model's apparent calibration performance on the training data, while the bias-corrected line represents the more reliable calibration curve after statistical adjustment. The closer the corrected curve is to the Ideal line, the better the model’s calibration. The *P*-value from the Hosmer–Lemeshow test is shown in the lower right corner of the chart. A *P*-value > 0.05 indicates no statistically significant deviation in the model’s calibration. As shown in the figure, both models are well-calibrated, and the combined model exhibits slightly better calibration accuracy in the high-risk region compared to the MCG model.

Supplementary Figure 2 Nomogram construction and calibration curve of the nomogram. (A): Risk of postoperative AF predicted by the MCG model; (C): risk of postoperative AF predicted by the combined model, which was constructed by adding three biochemical indicators (LDL, cTnI, and NT-proBNP) to the MCG model. (B), (D): The ideal line represents perfect calibration. The apparent line indicates the model's calibration performance on the training datasets, while the bias-corrected line shows the more reliable calibration curve after statistical adjustment. The closer the corrected curve is to the black ideal line, the better the model’s calibration. The *P*-value from the Hosmer–Lemeshow test is displayed in the lower right corner of the chart. A *P*-value > 0.05 indicates no statistically significant deviation in the model’s calibration. As shown in the chart, both models demonstrate good calibration, with the combined model showing slightly better calibration accuracy in the high-risk region compared to the MCG model.
